# The establishment of the first nonsurgical experimental model of progressive scoliosis -The biomechanical mechanism involved in the etiology of the thoracic scoliosis-

**DOI:** 10.1186/1748-7161-10-S1-O21

**Published:** 2015-01-19

**Authors:** Kensuke Kubota, Seiji Okada, Toshio Doi, Kazu Kobayakawa, Yoshihiro Matsumoto, Katsumi Harimaya, Takeshi Maeda, Yukihide Iwamoto

**Affiliations:** 1 Department of Orthopaedic Surgery, Graduate School of Medical Sciences, Kyushu University Fukuoka, Japan; 2 Department of Emergency & Critical Care Center, Kyushu University Hospital, Fukuoka, Japan; 3 Departments of Advanced Medical Initiatives, Graduate School of Medical Sciences, Kyushu University, Fukuoka, Japan; 4 Department of Orthopaedic Surgery, Kyushu University Beppu Hospital, Oita, Japan; 5 Department of Orthopaedic Surgery, Spinal Injuries Center, Fukuoka, Japan

## Objective

Idiopathic scoliosis is a structural, lateral, rotated curvature of the spine. Scoliosis may worsen progressively, in some cases leading to back pain, body image concerns, and cardiopulmonary compromise. To date, a variety of pathogenic factors have been proposed as a cause of idiopathic scoliosis. However, the pathogenesis of idiopathic scoliosis remains unclear, and a consistent and relevant animal model has not yet been established. We recently reported that the anteroposterior chest dimension in patients with thoracic scoliosis was significantly smaller than that in normal subjects and that there was a correlation between the anteroposterior chest dimension and the severity of the thoracic curvature. These findings led us to hypothesize that rib cage deformities were associated with the etiology of the thoracic scoliosis. The goal of this study was to clarify whether a shallow chest depth is a causative factor or a consequence of structural scoliosis, and also to establish a nonsurgical mouse model of progressive scoliosis.

## Material and methods

To examine the relationship between ribcage development and the pathomechanism underlying progressive scoliosis, a plastic restraint limiting anteroposterior ribcage development was braced on the chest of four-week-old mice. All study mice underwent whole spine radiographs, and the severity of scoliosis was consecutively measured with Cobb's angle. The ribcage rotation angle and anteroposterior chest dimension were measured by micro-computed tomography (CT) scanning, and the relationship between these factors and Cobb's angle was examined. To examine whether the imbalanced load transmission through the ribs to the vertebral body was involved in our model, we performed rib neck osteotomy in the mice.

## Results

The thoracic restraint did not provoke spinal curvature immediately after it was applied, but the mice gradually developed progressive scoliosis. After consecutive wearing the restraint, radiographic and CT images exhibited the existence of a right thoracic curvature, right vertebral rotation, and ribcage deformity in the mice. The anteroposterior chest dimension was statistically correlated with both Cobb's angle and the ribcage rotation angle. The progression of spinal deformity was observed only during the adolescent growth spurt and plateaued thereafter. The left-side osteotomy led to the development of progressive left thoracic curvature, while the bilateral surgery provoked no spinal scoliosis, even with the restraint.

## Conclusions

We successfully established the first non-surgical experimental model of progressive scoliosis, and also demonstrated that a ribcage deformity with an imbalanced load to the vertebral body resulted in progressive structural scoliosis.

